# Hyperkalemia and severe rhabdomyolysis

**DOI:** 10.3402/jchimp.v1i3.8707

**Published:** 2011-10-17

**Authors:** Marc Mugmon

**Affiliations:** Department of Medicine, Union Memorial Hospital, Baltimore, MD, USA

A 47-year-old homeless man with a history of bipolar disorder and polysubstance abuse was found under a bridge with altered mental status and severe left calf pain. Evaluation in the emergency department revealed a cachectic, pale man with a blood pressure of 132/84, pulse 74, and respirations 18. He was afebrile. Oxygen saturation was 98% on room air. Examination revealed marked swelling of the left leg, which was also pulseless. A compartment syndrome was diagnosed. Urgent fasciotomy was performed, and the patient was treated in the critical care unit. After a long hospital course he was discharged after 22 days.

**Fig. 1:**
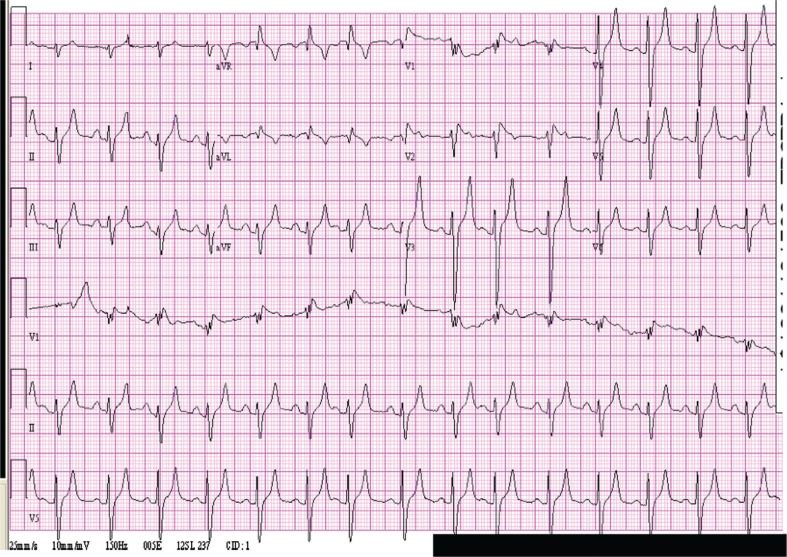
Electrocardiogram while potassium was 8.0 demonstrating tall, peaked T waves and wide QRS.

The initial electrocardiogram (ECG) (Fig. 1) revealed tall, peaked T waves (greater than 10 mm) and a QRS duration of 100 msec. Creatine phosphokinase (CK) initially was 67,762 units/L and increased to 149,440 units/L, indicative of severe rhabdomyolysis. Creatinine increased to 11, and emergency hemodialysis was undertaken. CK eventually dropped to normal after 14 days.

**Fig. 2:**
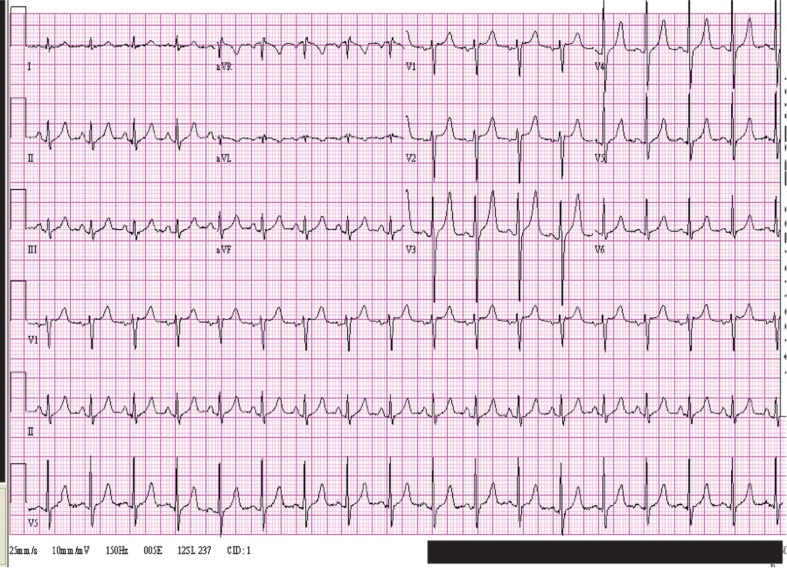
Electrocardiogram while potassium was 5.9 demonstrating narrowing of the QRS and less peaked T waves.

**Fig. 3:**
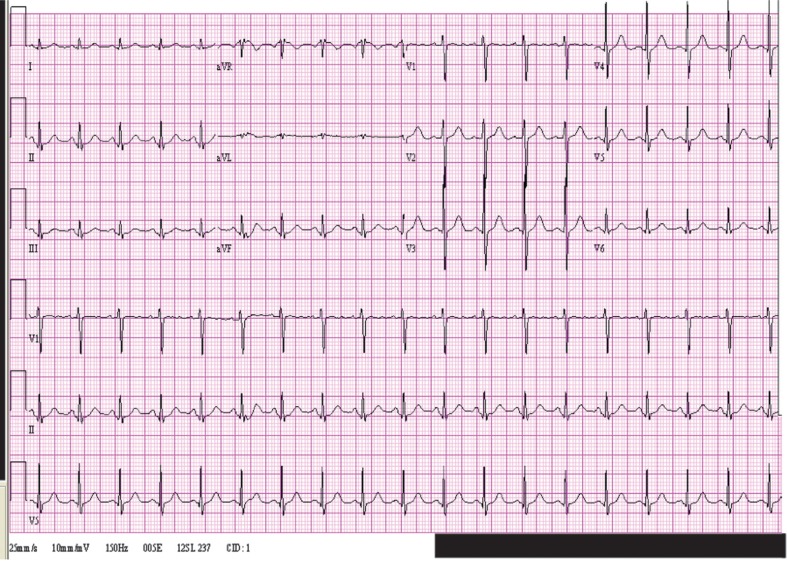
Normal electrocardiogram when potassium normalized.

The electrocardiographic changes were typical of hyperkalemia, and the initial potassium was 8.0. A follow-up tracing taken soon after the administration of calcium chloride, sodium bicarbonate, and sodium polystyrene sulfonate showed narrowing of the QRS duration to 80 msec, and the T waves were not as peaked but were still prominent. (Fig. 2) Potassium had dropped to 5.9 by that time. Potassium decreased further to 4.2 after 9 more hours, and the tracing at that time was identical to the normal one taken a week later (Fig. 3).

Hyperkalemia is potentially life threatening by virtue of its effects on cardiac rhythm and function (1). The mortality rate of severe hyperkalemia not treated rapidly is 67% (2). Potassium levels of 5.5–6.0 are defined as mild, 6.1–7.0 moderate, and greater than 7.0 severe.

The ECG findings in hyperkalemia generally follow a progression. The first manifestations are peaked or tented T waves, especially in the precordial leads. This may be accompanied by P wave flattening. As hyperkalemia becomes moderate, the PR interval may increase and the P waves may disappear, with a subsequent junctional rhythm. QRS widening and axis shifts may develop as hyperkalemia progresses. More severe degrees lead to bizarre widening of the QRS and the development of a sine wave appearance, followed by ventricular fibrillation or asystole (3).

Treatment should be directed towards stabilizing the heart and trying to move extracellular potassium to the intracellular compartment. Additionally, eliminating further sources of potassium is important. Calcium chloride restores the normal gradient between the threshold and resting potential. Its onset of action is 5 min or less and may last for 60 min. It is usually not needed when the only ECG manifestation is a peaked T wave. Glucose and insulin promote potassium shift into cells, generally within 30 min. Sodium bicarbonate may be useful if acidosis is present, by raising pH and promoting potassium movement into cells. Albuterol may also be useful by increasing insulin concentration and may be helpful when fluid overload is a concern in the presence of renal failure. Diuretics may be effective in patients who have adequate renal function. Binding resins exchange potassium for sodium in the GI tract. Finally, dialysis may be necessary especially if there is concern of ongoing potassium being released, due to severe muscle injury, as seen in this patient.
